# Effects of β-amyloid (1-42) Administration on the Main Neurogenic Niches of the Adult Brain: Amyloid-Induced Neurodegeneration Influences Neurogenesis

**DOI:** 10.3390/ijms232315444

**Published:** 2022-12-06

**Authors:** Konstantin Yenkoyan, Tigran Margaryan, Senik Matinyan, Vergine Chavushyan, Margarita Danielyan, Tigran Davtyan, Michail Aghajanov

**Affiliations:** 1 Neuroscience Laboratory, Cobrain Center, Yerevan State Medical University after M. Heratsi, Yerevan 0025, Armenia; 2 Department of Biochemistry, Yerevan State Medical University after M. Heratsi, Yerevan 0025, Armenia; 3 Laboratory of Neuroendocrine Relations, L.A. Orbeli Institute of Physiology of NAS, Yerevan 0028, Armenia; 4 Laboratory of Histochemistry and Electromicroscopy, L.A. Orbeli Institute of Physiology of NAS RA, Yerevan 0028, Armenia; 5 Analytical Laboratory of Scientific Centre of Drug and Medical Technology Expertise JSC, Yerevan 0051, Armenia

**Keywords:** Alzheimer’s disease, amyloid, neurogenesis, neurogenic niches, adult brain

## Abstract

Alzheimer’s disease (AD) is the most prevalent neurodegenerative disorder and warrants further study as well as timely treatment. Additionally, the mechanisms of the brain’s intrinsic defense against chronic injury are not yet fully understood. Herein, we examined the response of the main neurogenic niches to amyloid exposure and the associated changes in structure and synaptic activity. Flow cytometry of Nestin-, Vimentin-, Nestin/Vimentin-, NeuN-, GFAP-, NeuN/GFAP-, NSE-, BrdU-, Wnt-, BrdU/Wnt-, VEGF-, Sox14-, VEGF/Sox14-, Sox10-, Sox2-, Sox10/Sox2-, Bax-, and Bcl-xL-positive cells was performed in the subventricular zone (SVZ), hippocampus, and cerebral cortex of rat brains on 90th day after intracerebroventricular (i.c.v.) single injection of a fraction of β-amyloid (Aβ) (1-42). The relative structural changes in these areas and disruptions to synaptic activity in the entorhinal cortex–hippocampus circuit were also evaluated. Our flow analyses revealed a reduction in the numbers of Nestin-, Vimentin-, and Nestin/Vimentin-positive cells in neurogenic niches and the olfactory bulb. These changes were accompanied by an increased number of BrdU-positive cells in the hippocampus and SVZ. The latter changes were strongly correlated with changes in the numbers of VEGF- and VEGF/Sox14-positive cells. The morphological changes were characterized by significant neural loss, a characteristic shift in entorhinal cortex–hippocampus circuit activity, and decreased spontaneous alternation in a behavioral test. We conclude that although an injection of Aβ (1-42) induced stem cell proliferation and triggered neurogenesis at a certain stage, this process was incomplete and led to neural stem cell immaturity. We propose the idea of enhancing adult neurogenesis as a promising strategy for preventing dementia at healthy elderly people andpeople at high risk for developing AD, or treating patients diagnosed with AD.

## 1. Introduction

Alzheimer’s disease (AD) is the most prevalent neurodegenerative disorder. While the incidence of this disease is predicted to increase dramatically, therapeutic modalities that can sufficiently affect the course of the disease are lacking [1]. Considering the pathogenesis, the therapeutic approaches for AD are focused on counterbalancing neurotransmitter disturbance, anti-amyloid therapy, anti-tau therapy, anti-neuroinflammatory therapy, the inhibition of oxidative injury, and brain stimulation [2,3]. However, among all the mentioned treatments, today, only acetylcholinesterase inhibitors [4], *N*-methyl-d-aspartate receptor antagonist [5], and one representative from anti-Aβ-monoclonal antibodies are approved by the FDA [6]. Despite the latest promising findings of a phase 3 study on the efficacy of aducanumab (anti-Aβ monoclonal antibody) as a therapy for early-stage Alzheimer’s disease, which slows cognitive decline [7], the existing treatments are still unable to achieve clinically meaningful benefits. The explanation for this may be continuous neuronal damage, synaptic dysfunction, and the massive loss of synapses [8]. The outstanding questions are as follows: (1) which mechanisms are involved in chronic brain damage, (2) and to what extent do these mechanisms contribute to alleviating neurodegeneration-induced changes? In this context, the functional significance of adult neurogenesis acquires additional importance [9]. The generation of new neurons from neural precursor cells (NPCs) is a multi-step process that includes the proliferation of NPCs, fate determination, migration, and neural maturation. Various experiments have identified the main areas of neurogenesis in the mammalian central nervous system under normal conditions [10] and in cases of brain damage [11,12]. These areas include the anterior part of the subventricular zone (SVZ) along the lateral ventricles and the subgranular zone (SGZ) of the dentate gyrus (DG) of the hippocampus [12]. Immature neurons migrate from the SVZ via the rostral migratory stream to the olfactory bulb, but only a few of them survive and acquire adult neural properties [13]. Hippocampal neurogenesis is highly dependent on several environmental and cell-intrinsic factors and can be linked to specific physiological functions [14]. However, it remains unclear whether these mechanisms are altered during neurodegeneration, or how AD influences this process. Several animal models of AD demonstrated decreased neurogenesis in both the DG and the SVZ in amyloid-induced neurodegeneration [15,16]. In contrast, an increase in neurogenesis was observed following in vitro exposure to β-amyloid (Aβ) 1-42 [17], but the administration of an Aβ (25-35) fraction decreased the number of BrdU-, NeuN-, and DCX-positive cells in the hippocampal granule cell layer [18]. Although the experimental conditions largely differed, these findings revealed altered and dysfunctional neurogenesis in both the SVZ and SGZ of the DG [19].

Given the current understanding of neurodegeneration, newly born immature neurons may be closely associated with the remodeling of vasculature within the neurovascular units (NVUs). Amyloidogenesis alone activates a chain of reactions leading to hypervascularization, increased vascular permeability [20], and the redistribution of tight junctions [21]. However, the generation of new vasculature facilitates highly coupled neurorestorative processes, including neurogenesis and synaptogenesis, which, in turn, lead to improved functional recovery.

Thus, accumulating evidence suggests that a complete understanding of neurodegeneration-induced neurogenesis in the adult brain is needed; therefore, the examination of this aspect was selected as the aim of the current study. We utilized an Aβ (1-42)-induced model of AD-like pathology and investigated characteristic morphological changes and electrophysiological recordings from single neurons of the hippocampus under high-frequency stimulation of the entorhinal cortex.

## 2. Results

### 2.1. Flow Cytometry Results

#### 2.1.1. Hippocampus

**Figure 1:**
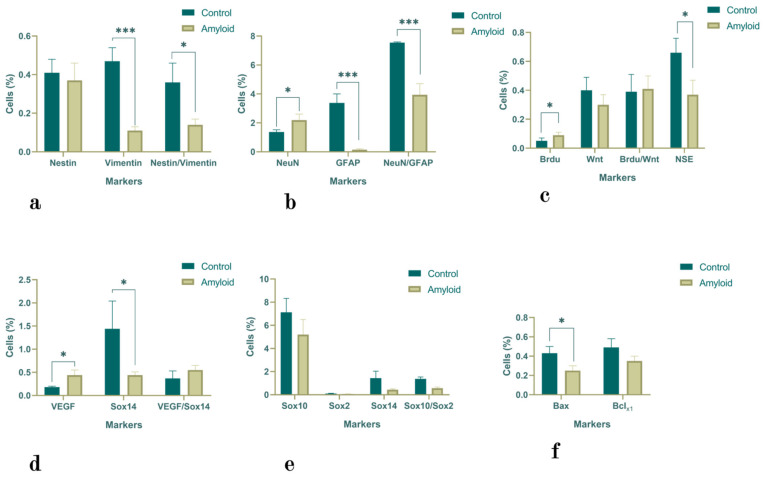
The proportion of Nestin-, Vimentin-, and Nestin/Vimentin- (**a**); NeuN-, GFAP-, and NeuN/GFAP- (**b**); NSE-, BrdU-, Wnt-, and BrdU/Wnt- (**c**); VEGF-, Sox14-, and VEGF/Sox14- (**d**); Sox10-, Sox2-, Sox14-, and Sox10/Sox2- (**e**); and Bax-and Bcl-xL-positive (**f**) cells in the hippocampus under normal conditions and under the influence of Aβ (1-42). Statistical significance is indicated as follows: *** *p* < 0.0001 and * *p* < 0.05.

Although there was no significant difference in the number of Nestin-positive cells in the hippocampus after the i.c.v. administration of Aβ (1-42), the numbers of Vimentin (*p* < 0.0001) and Nestin/Vimentin (*p* < 0.05) double-positive cells significantly decreased (4.27- and 2.57-fold decreases, respectively) (Figure 1a). There was a 1.6-fold increase in the number of NeuN-positive cells (*p* < 0.05) and a 22.53-fold decrease in the number of GFAP-positive cells (*p* < 0.0001). The number of NeuN/GFAP double-positive cells also decreased (by 1.91-fold; *p* < 0.0001) (Figure 1b). The administration of Aβ (1-42) failed to alter the numbers of Wnt-positive and BrdU/Wnt double-positive cells, although it led to a decreased number of NSE-positive cells (1.78-fold; *p <* 0.05) and an increased number of BrdU-positive cells (1.8-fold; *p* < 0.05) (Figure 1c). The numbers of VEGF- (*p* < 0.05) and VEGF/Sox14-positive cells also increased (Figure 1d). The numbers of Sox10-, Sox2-, and Sox10/Sox2-positive cells decreased by 1.37-, 1.66-, and 2.4-fold, respectively, although these values were not significantly different from those of the control group (Figure 1e). The same tendency was observed in terms of the numbers of Bax- (*p* < 0.05) and Bcl-xL-positive cells (with 1.72- and 1.4-fold decreases from the control levels, respectively) (Figure 1f).

#### 2.1.2. Subventricular Zone

**Figure 2:**
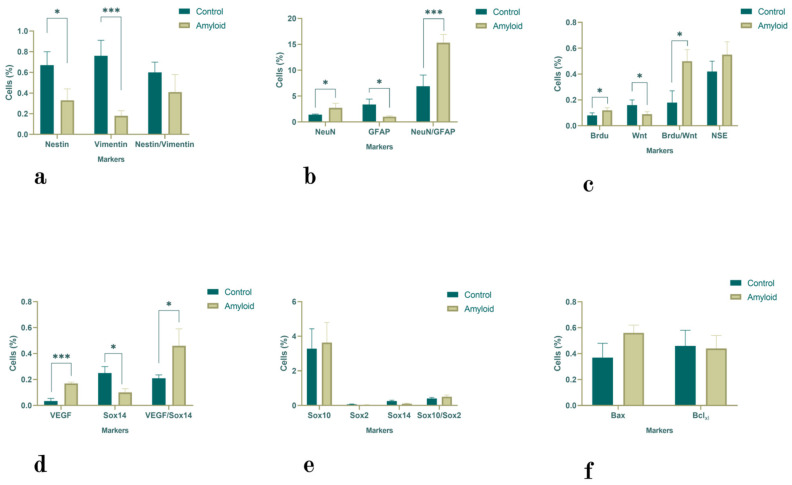
The proportions of Nestin-, Vimentin-, and Nestin/Vimentin- (**a**); NeuN-, GFAP-, and NeuN/GFAP- (**b**); NSE-, BrdU-, Wnt-, and BrdU/Wnt- (**c**); VEGF-, Sox14-, and VEGF/Sox14- (**d**); Sox10-, Sox2-, Sox14-, and Sox10/Sox2- (**e**); and Bax- and Bcl-xL-positive cells (**f**) in the subventricular zone under normal conditions and with Aβ (1-42). Statistical significance is indicated as follows: *** *p* < 0.0001 and * *p* < 0.0.

The administration of Aβ (1-42) reduced the number of Nestin- (2.03-fold; *p* < 0.05) and Vimentin-positive cells (4.22-fold; *p <* 0.0001) (Figure 2a). The number of GFAP-positive (3.3-fold; *p* < 0.05) cells was also reduced in comparison with the control levels. However, the numbers of NeuN-positive (1.95-fold; *p <* 0.05) and NeuN/GFAP double-positive cells (2.2-fold; *p* < 0.0001) increased after amyloid exposure (Figure 2b). The numbers of NSE- and BrdU-positive cells as well as the number of BrdU/Wnt double-positive cells showed a tendency to increase after amyloid exposure (by 1.33-fold, 1.77-fold, and 2.77-fold, respectively), although only the last two changes were statistically significant (*p* < 0.05) (Figure 2c). Additionally, we detected a 5-fold increase in the number of VEGF-positive cells (*p* < 0.0001) and a 2.2-fold increase in the number of VEGF/Sox14 double-positive cells (*p* < 0.05) (Figure 2d). However, the number of Sox14-positive cells decreased (*p* < 0.05). The changes in the numbers of Sox10-, Sox2-, Sox10/Sox2-, and Bcl-xL-positive cells were not statistically significant, although there was a 1.5-fold increase in Bax-positive cells (Figure 2e,f).

#### 2.1.3. Olfactory Bulb

**Figure 3:**
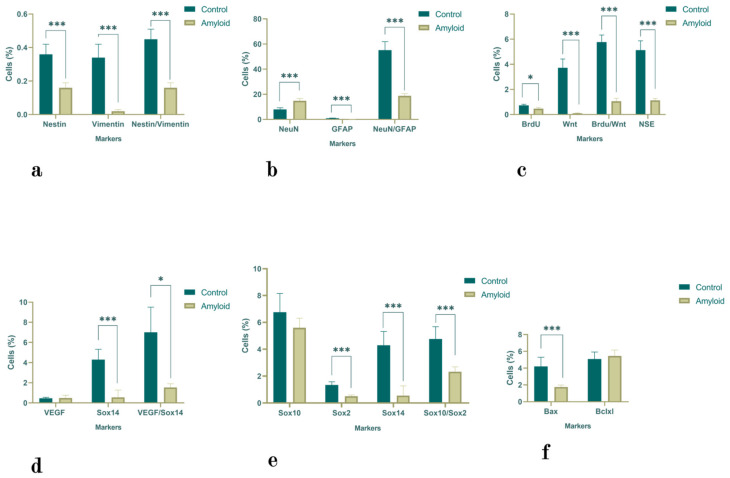
The proportion of Nestin-, Vimentin-, and Nestin/Vimentin- (**a**); NeuN-, GFAP-, and NeuN/GFAP- (**b**); NSE-, BrdU-, Wnt-, and BrdU/Wnt- (**c**); VEGF-, Sox14-, and VEGF/Sox14- (**d**); Sox10-, Sox2-, Sox14-, and Sox10/Sox2- (**e**); and Bax-and Bcl-xL-positive cells (**f**) in the olfactory bulb under normal conditions and with Aβ (1-42). Statistical significance is indicated as follows: *** *p <* 0.0001 and * *p <* 0.05.

The numbers of Nestin- and Vimentin-positive cells as well as the number of Nestin/Vimentin double-positive cells decreased after amyloid exposure (*p* < 0.0001) (Figure 3a). A similar tendency was observed in the numbers of GFAP-positive and NeuN/GFAP-positive cells, in which the latter decreased by 4.54-fold (*p* < 0.0001). In contrast, the number of NeuN-positive cells was increased (1.85-fold; *p <* 0.0001) (Figure 3b). The numbers of NSE- (4.54-fold; *p <* 0.0001), BrdU- (1.55-fold; *p <* 0.05), and Wnt (37.2-fold; *p <* 0.0001)-positive cells as well as the number of BrdU/Wnt (4.55-fold; *p <* 0.0001)-double-positive cells decreased compared with the control values (Figure 3c). The changes in the numbers of VEGF-, Sox10-, and Bcl-xL-positive cells were not significant (Figure 3d–f). However, there was a 7.88-fold decrease in the number of Sox14 cells (*p <* 0.0001) and a 4.55-fold decrease in the number of VEGF/Sox14-double-positive cells (*p <* 0.05) (Figure 3d). The number of Sox2-positive cells decreased by 2.68-fold (*p <* 0.0001), and the number of Sox10/Sox2 double-positive cells decreased by 2-fold (*p <* 0.05) (Figure 3e). A similar tendency was observed in the number of Bax-positive cells (2.4-fold; *p <* 0.05) (Figure 3f).

### 2.2. Morphological Changes

#### 2.2.1. Subventricular Zone

**Figure 4:**
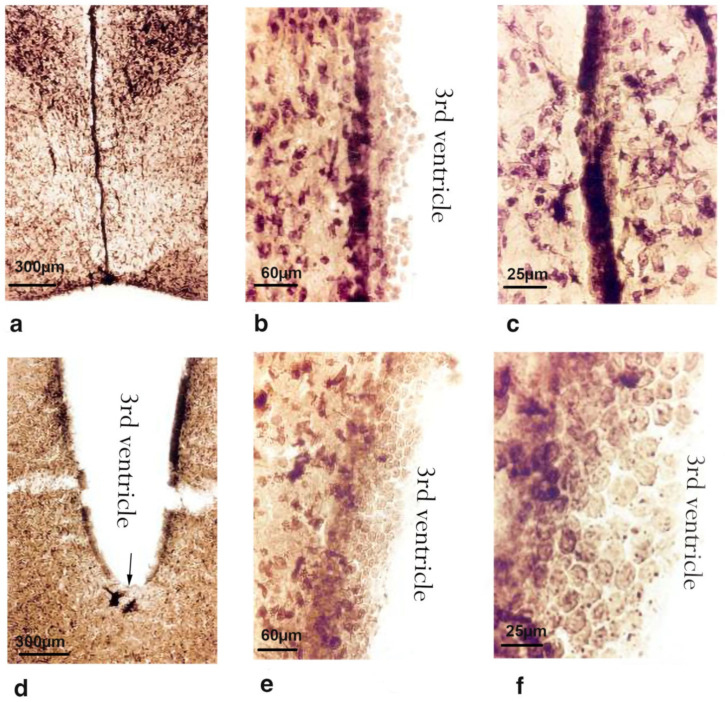
Microphotographs of rats’ brain sections of the 3rd ventricle: (**a**–**c**) control animals; (**d**–**f**) animals under the influence of bilateral single i.c.v. administration of Aβ (1-42). Magnification: 25× (**a**,**d**); 160× (**b**,**e**); 400× (**c**); 1000× (**f**).

In the control rats, the walls of the brain’s third ventricle are in close contact, and the expanded area is represented by a continuous cylindrical epithelial lining of ependymal cells (Figure 4a–c). In some places, these cells were atypically found in several layers (Figure 4b). These cells are in close contact in the control rats and exhibit higher phosphatase activity than neurons and glial cells. A large number of thin processes connected the nerve cells to the ependymal cells (Figure 4c). The granular sediment in the soma may have been the product of increased secretions from ependymal cells. The sites indicated in Figure 4 are likely the areas where neural cells contact the ependymal layer, revealing existing connections. In the rats administered bilateral i.c.v. injections of the Aβ peptide, sites of desquamation (Figure 4d) and the multilayer proliferation of ependymal cells in the form of round balls (Figure 4e,f) were observed.

#### 2.2.2. Cerebral Cortex and Hippocampus

**Figure 5:**
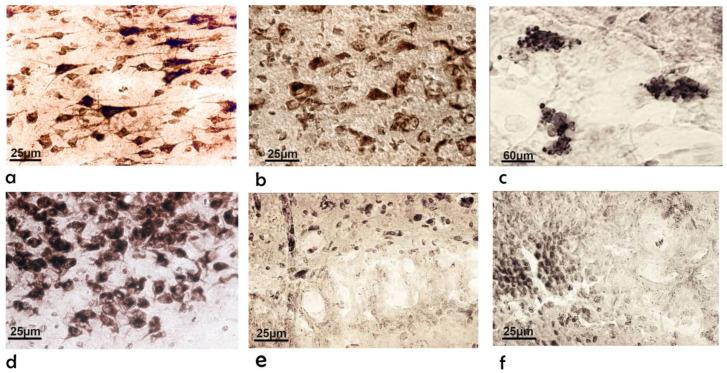
Microphotographs of rats’ brain sections: (**a**–**c**) cerebral cortex, (**d**–**f**) hippocampus, (**a**,**d**) of intact animals; (**b**,**c**,**e**,**f**) animals under the influence of bilateral single i.c.v. administration of Aβ (1-42). Magnification: 400× (**a**,**b**,**d**–**f**); 160× (**c**).

The main characteristic of the frontal sections of the cortex in the control rats was the intensity of staining in the pyramidal cells. The conical body of the pyramidal cells became a thick, radially directed dendrite (Figure 5a). The greatest phosphatase activity was observed at the site where the primary dendrite originated. Several short and thin lateral dendrites deviated from the circumference of the base of each pyramidal neuron. Usually, the axon starts in the middle of the base and reaches down to the white matter. The hippocampal neurons of the control rats differed in size, shape, and branching architecture (Figure 5d). Intensely colored large granules were observed in the bodies and processes of hippocampal cells (Figure 5d).

After Aβ (1-42) exposure, neurons in all layers of the cerebral cortex lost their characteristic shape and became rounded and swollen. The branching pattern was lost, and central chromatolysis was observed (Figure 5b). We also noticed single, shapeless, dark-colored formations against the background of a complete absence of cellular reactions (Figure 5c). The matrix of the brain architecture in the images below was not filled due to the lack of reaction with glial cell nuclei. We observed single, circular neurons in some areas of the CA1 hippocampal field as well as areas with a complete absence of neurons (Figure 5e,f). The neurons in the CA3 field and DG were more resistant to amyloid exposure.

### 2.3. Behavioral Results

**Figure 6:**
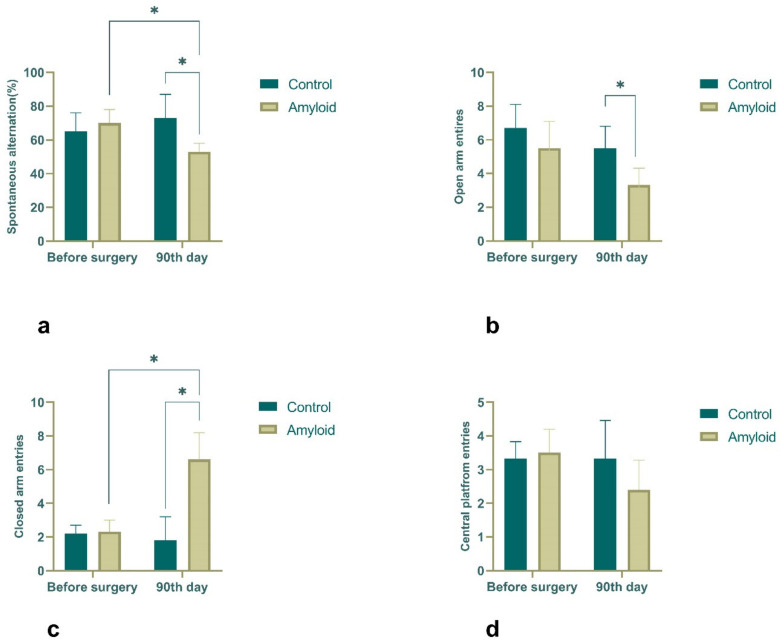
Y-maze (**a**) and elevated plus maze (**b**–**d**) test results: spontaneous alternation in the control and experimental groups before and after 90 days of amyloid exposure (**a**); counts of open arm entries (**b**), closed arm entries (**c**), and central platform crossings (**d**) before and after 90 days of amyloid single exposure. Statistical significance is indicated as follows: * *p <* 0.05.

Regarding the cognitive abilities of the rats, which were tested in a Y maze, it was observed that in the experimental group, the spontaneous alternation score was significantly decreased after 90 days of amyloid exposure compared to the baseline level (70 ± 8% vs. 53 ± 5%; *p <* 0.05) (Figure 6a). A more pronounced decrease in spontaneous alternation was fixed when comparing the changes between Aβ (1-42)-injected and vehicle-treated groups on the 90^th^ day after surgery (73 ± 14% vs. 53 ± 5%; *p <* 0.05) (Figure 6a). The anxiety levels of the rats tested in the elevated plus maze after 90 days of amyloid exposure demonstrated that the amyloid group animals preferred the closed arms of the maze compared to the control group (2.3 ± 0.7 vs. 6.6 ± 1.60; *p <* 0.05), although there was no significant difference in the number of times, they crossed the central platform (3.5 ± 0.7 vs. 2.4 ± 0.88) (Figure 6b–d). The obtained data suggested elevated levels of anxiety in the amyloid-injected rats compared to the controls.

### 2.4. Electrophysiological Recordings

**Figure 7:**
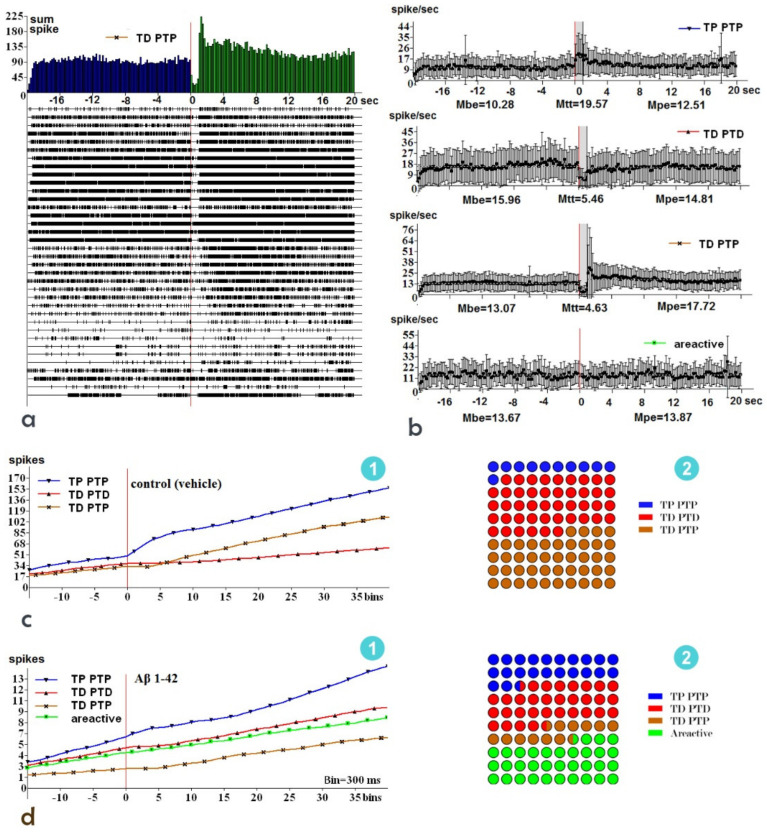
Peristimulus activity of hippocampal neural populations displaying TD-PTP, presented as a raster plot, and the sum of spikes for these neurons (**a**). The mean frequency histogram with processed data displaying activity during the peristimulus (M_be_) and post-stimulus events (M_pe_) as well as during high-frequency stimulation (tetanization time, M_tt_), indicating the response type (**b**). The averaged cumulative histograms for hippocampal neural populations with specific response types in the control (**c1**) and experimental groups (**d1**). The distribution of response types in mentioned control (**c2**) and experimental (**d2**) groups respectively.

In the in vivo extracellular electrophysiological recordings, the spike activity of a single neuron was recorded in real time during the 20 s before stimulation (background activity), during HFS (1 s), and during the 20 s after stimulation (post-stimulus activity); the activity varied depending on the response type. Then, the software grouped neurons by response types and averaged the group data. We constructed peristimulus histograms of mean frequency (Figure 7b) and cumulative histograms (Figure 7c,d). The peristimulus activity for a single hippocampal neuron showing TD-PTP to HFS of the entorhinal cortex is shown in Figure 7a; the number of spikes is shown as a raster plot. The numerical values of the mean frequency (spike/s) of spike activity before stimulation (M_BE_), after stimulation (M_PE_), and during high-frequency stimulation (tetanization time, M_HFS_) are presented in the raster plot, as well as the responses of neural populations exhibiting TP-PTP, TD-PTD, or no response (Figure 7).

Analysis of the cumulative histograms revealed that the experimental group exhibited the highest (quantitative) rates of pre- and post-stimulus spikes in the neurons displaying the TP-PTP response, the lowest rates in the neurons displaying TD-PTP, and approximately equivalent rates in the nonreactive neurons as well as those displaying TD-PTD (Figure 7d). In the control group (n = 294 neurons), the neurons displaying TD-PTP and TD-PTD exhibited almost identical rates of peristimulus spikes (background activity); this level of activity was significantly lower than that of the neurons displaying TP-PTP (Figure 7c). In the control group, an analysis of the synaptic activity of neural units in the hippocampal CA1 field, which reflects the functional activity of the CA3 field and DG, revealed the following proportion of response types: 42.67% TD-PTD (132 of 294 neurons), 40.76% TD-PTP (130 of 294 neurons), and 16.56% TP-PTP (32 of 294 neurons) (Figure 7(c2)). In the experimental group, the neurons exhibited the following proportion of response types: 37.17% TD-PTD (42 of 113 neurons), 14.16% TD-PTP (16 of 113 neurons), and 26.55% TP-PTP (30 of 113 neurons). A total of 44 of 113 neurons (38.94%) were nonreactive (Figure 7(d2)). Figure 7c, d show a slight, but significant, difference in the percentage of neurons displaying TD-PTD between the control and experimental groups (42.67% and 37.17%, respectively; *p <* 0.005), which suggests that the population of neurons displaying TD-PTD are the least vulnerable to the effects of Aβ (1-42). Regarding the populations of neurons displaying TP-PTP, we observed similar differences: 26.55% in the experimental group versus 16.56% in the control group (*p <* 0.005).

## 3. Discussion

**Figure 8:**
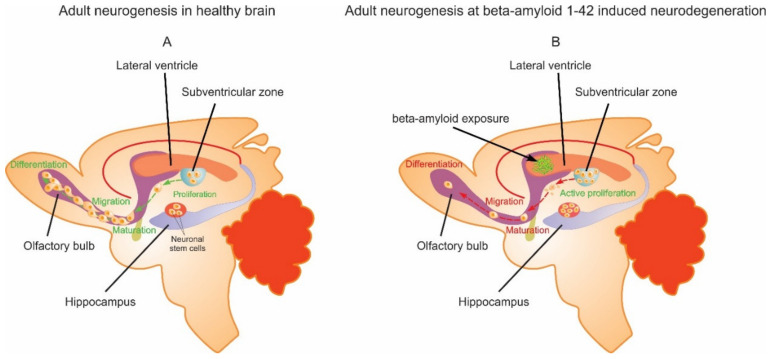
(**A**) Adult neurogenesis in the brains of healthy rats. Here, we depict neurogenesis under normal conditions: stem cell proliferation in neurogenic niches (the SVZ and hippocampus), differentiation and migration, maturation, and finally integration into adjacent neural circuits. (**B**) Adult neurogenesis in the brain after aggregated Aβ (1-42) single exposure. Amyloid exposure resulted in increased stem cell proliferation in neurogenic niches (the SVZ and hippocampus), followed by impaired migration and incomplete maturation.

We found a reduction in the numbers of Nestin-, Vimentin-, Nestin/Vimentin-, and Sox 2-positive cells in the olfactory bulb, hippocampus, and SVZ after amyloid exposure, although in some cases, the reduction was not significant. Neurodegeneration appears to lead to neural stem cell deficiency in adult brains, disturbing the initial process of neurogenesis. However, we observed an increase in the number of BrdU-positive cells in the hippocampus and SVZ (but not the olfactory bulb); these two regions are the main neurogenic niches of the brain [22]. Notably, the significant increase in the number of BrdU-positive cells was accompanied by a significant increase in the number of BrdU/Wnt-double-positive cells in the SVZ. As BrdU indicates the S-phase of mitosis and cell proliferation and the Wnt/β-catenin pathway is responsible for regulating neural stem cell proliferation and self-renewal [20,23,24], Aβ(1-42)-induced neurodegeneration may stimulate the proliferation of stem cells in the SVZ of the adult brain and then help to regulate the process (Figure 8).

In addition, changes in the number of BrdU-positive cells were correlated with increases in the numbers of VEGF- and VEGF/Sox14-positive cells in the SVZ and hippocampus. The quantities of these cells, except for the VEGF-positive cells, were correspondingly decreased in the olfactory bulb. These findings highlight the importance of cross-talk between the processes of neurogenesis and angiogenesis and are consistent with data from other groups, who found that changes in neurogenesis often occur in parallel to changes in angiogenesis [25]. These processes share modulating factors, including VEGF and its receptor Flk-1; thus, the increased numbers of NeuN-, NeuN/GFAP-, and NSE-positive cells in the SVZ may be a direct consequence of the aforementioned changes. However, although the numbers of these cells increased, they did not undergo changes proportional to those of the Sox10/Sox2-positive cells and the NSE-positive cells, whose quantities were altered in the SVZ as well as in the hippocampus and olfactory bulb.

Although such multifaceted changes make it difficult to reach a complete conclusion, it is clear that neurodegeneration stimulates neurogenesis in the SVZ and, to a lesser extent, in the hippocampus. However, it remains unclear whether these stem cell reactions can contribute to recovery from neurodegeneration [26]. Although the SVZ increases neurogenesis and serves as a reservoir of newly generated neural stem cells, the transformation of initial progenitor cells into mature neurons, astrocytes, and oligodendrocytes was not complete (Figure 8). This finding was also confirmed by the decreased number of GFAP-positive cells in all the investigated brain structures and the decreased number of NeuN/GFAP-positive cells in the hippocampus and olfactory bulb after amyloid exposure. Considering the olfactory bulb, which is the main site of migrating neural stem cells [27], we noted a decreased number of cells with mature neural and glial markers, which supports the idea that amyloid-induced neurogenesis is defective; even if these neural progenitor cells successfully migrate, some do not mature. In our opinion, such neural stem cell immaturity is due to the failure of internal factors, which regulate the genesis and terminal differentiation of neurons and glia in order to support stem cells. Our previous experiments revealed ambiguous shifts in the levels of nerve growth factor (NGF) and insulin-like growth factor (IGF) in brain structures; Aβ (25-35) induced an increase in IGF-1 levels in the hippocampus and cerebral cortex. Although NGF levels slightly increased in the hippocampus, these levels decreased in the cerebral cortex, indicating that sufficient concentrations of growth factors are a prerequisite for neural maturation [28,29]. The ratio of Bax-/Bcl-xL-positive cells increased by 50% in the SVZ and decreased 2.5-fold in the olfactory bulb, indicating the activation of the anti-apoptotic cascade. However, this does not exclude neural death caused by an apoptotic pathway, for example, at the level of annexin or caspases. In contrast to our findings, previous studies have found that reduced levels of NGF and BDNF during neurodegeneration promoted caspase activation in neural stem cells by activating the PI3K/Akt and mitogen-activated protein kinase (MAPK) pathways [30].

The population of neurons exhibiting TD-PTP and nonreactive responses suggests that in the experimental group, the relative proportion of nonreactive neurons was increased due to a reduction in the number of neurons displaying TD-PTP. Almost half of the recorded neurons showed decreased responses during HFS of the entorhinal cortex (TD), and a large portion of hippocampal neurons (38.94%) were nonreactive, which confirmed our morphological findings regarding neurodegeneration. Changes in the ratios of the considered parameters caused by neural activity might have resulted from exposure to Aβ (1-42), thereby altering the structural organization of entorhinal cortex–hippocampus connections and the biochemical/biophysical indices of synapses; such neural changes were associated with behavioral changes in spontaneous alternation after 90 days of amyloid exposure. The preference of amyloid group animals for the closed arms increased after amyloid exposure, suggesting altered anxiety levels. The locomotor activity, calculated as the total number of entries, did not change significantly. However, animals from the amyloid group tended to make wrong decisions in the case of the Y maze and preferred closed arms in the case of the elevated plus maze. Thus, cognitive decline and elevated anxiety were inherent to the Aβ (1-42)-injected rats. These outcomes are in line with our earlier results indicating a progressive decrease in spatial alteration starting from the 30th day of i.c.v. injection of Aβ (25-35) [31,32]. Thus, the current study revealed that acute brain exposure of Aβ (1-42) on the 90th day led to cognitive decline, elevated anxiety, aberrant spike activity of single hippocampal neurons evoked by the entorhinal cortex, damage to the neurons in all layers of the cerebral cortex and regions of hippocampus (CA1 region to the greatest extent), and morphologically detected multilayer proliferation of ependymal cells, which was confirmed by the increase in the number of BrdU-positive cells in both the SVZ and the hippocampus. Thus, together with our previous findings [31,33,34,35], the spatiotemporal pattern of changes in synaptic plasticity, accompanied by neural loss and the incomplete response of neurogenic niches, reveal the complexity of amyloid-induced neurodegeneration and indicate the possibility of developing effective neuroprotective agents that modulate amyloid-induced neurodegeneration as well as neuro- and angiogenesis.

### Limitations of This Study

As the factors included in this analysis cover a wide range of possible mechanisms, we limited this study to showing the response of the main neurogenic niches and accompanying morphological and electrophysiological changes. The repertoire of markers included in this analysis indicates many possibilities for exploring the molecular mechanisms of amyloid-induced neurogenesis alterations and associated growth factor changes in future investigations.

## 4. Methods and Materials

### 4.1. Animals

Experiments were performed on 30 mature white Sprague–Dawley male rats at 14–15 months of age. Animals weighing 220–300 g were kept under standard laboratory conditions in a vivarium. The experiments were carried out following the European Communities Council Directive (86/609/EEC) on the care and use of animals for experimental procedures; the protocol was approved by the Institutional Animal Care and Use Committee.

### 4.2. Experimental Protocol

The animals were divided into 2 groups: the control group, which received a single vehicle treatment, and the experimental group (the animal model of AD), which received single intracerebroventricular (i.c.v.) injection of aggregated Aβ (1-42). On 90th day after i.c.v. injection of Aβ, all animals (15 vehicle controls and 15 amyloid-injected) were tested in a battery of behavioral tests consisting of Y and elevated-plus mazes. After completion of the behavioral studies, 5 animals in each group were sacrificed, and Nestin-, Vimentin-, Nestin/Vimentin-, NeuN-, GFAP-, NeuN/GFAP-, NSE-, BrdU-, Wnt-, BrdU/Wnt-, VEGF-, Sox14-, VEGF/Sox14-, Sox10-, Sox2-, Sox10/Sox2-, Bax-, and Bcl-xL-positive cells were identified in the SVZ, hippocampus, and olfactory bulb via flow cytometry. Additionally, the structural changes in the same areas (3 animals per group) and disruption to synaptic activity in the entorhinal cortex–hippocampus circuit (7 animals per group) were examined.

### 4.3. Preparation of Aβ (1-42) Peptide

The peptide Aβ (1-42) was purchased from Sigma–Aldrich^®^ (St. Louis, MO, USA) and aggregated according to the manufacturer’s recommendations. The peptide was dissolved in sterile double-distilled water at a concentration of 1 mg/mL, aliquoted into tubes, and stored at −18 °C. It was “aged” by incubation at 37 °C for 96 h before the surgery to facilitate the formation of aggregates [36,37,38]. Light microscopy detected the existence of both birefringent fibril-like structures and globular aggregates. During fibril formation, several coexisting heterogeneous species are formed, and heterogeneity of the peptide samples creates some limits for the application of Aβ in rat models [39]. The aggregation of Aβ (1-42) was determined by a Thioflavin T (ThT)-binding assay (internal evaluation).

### 4.4. Surgical Procedure

The animals were anesthetized with Nembutal (40 mg/kg) and positioned in a stereotaxic frame; a midline sagittal incision was made in the scalp. Holes were drilled in the skull over the lateral ventricles using the following coordinates (according to the stereotaxic atlas of Paxinos and Watson [40]): 0.8 mm posterior to bregma and 1.5 mm lateral to the sagittal suture. Animals were injected once with 3 µL of sterile double-distilled water (control group) or 3 μg/3 µL of aggregated Aβ (1-42) solution (experimental group) in each cerebral lateral ventricle at a rate of 1 µL/min using the peristaltic pump.

### 4.5. Flow Cytometry of Cells

The selected brain regions (the hippocampus, SVZ, and olfactory bulb) were dissected according to the stereotaxic atlas of Paxinos and Watson [15], weighed, and homogenized on ice. Then, hippocampal, SVZ, and olfactory bulb cells were fixed in phosphate-buffered saline (PBS) containing 0.1% paraformaldehyde solution, 1% bovine serum albumin, and a cocktail of protease inhibitors (Roche Diagnostics, Mannheim, Germany) with pH = 7.4. For BrdU determination, rats received intraperitoneal (i.p.) injections of the nucleotide BrdU (50 mg/kg; Sigma-Aldrich^®^, Saint Louis, MO, USA), were sacrificed one hour later, and their target tissues were removed (anti-BrdU manufacter Chemicon^®^ International recommendation).

Fixed cells were incubated with BD FACS™ Permeabilizing Solution (BD Biosciences, San Jose, CA, USA) and intracellularly stained with primary monoclonal antibodies against Nestin (Abcam, Cambridge, UK), polyclonal Vimentin (Abcam, Cambridge, UK), polyclonal GFAP (Abcam, Cambridge, UK), monoclonal NeuN (Chemicon Int., Temecula, CA, USA), monoclonal BrdU (Chemicon^®^ Int., Temecula, CA, USA), polyclonal Wnt (Abcam, Cambridge, UK), polyclonal Sox2 (Abcam, Cambridge, UK), monoclonal VEGF (Abcam, Cambridge, UK), polyclonal Sox14 (Abcam, Cambridge, UK), monoclonal Sox10 (Abcam, Cambridge, UK), polyclonal NSE (Chemicon Int., Temecula, CA, USA), polyclonal Bax (Santa Cruz Biotechnology Inc., Dallas, TX, USA), and polyclonal Bcl-xL (Santa Cruz Biotechnology Inc., Dallas, TX, USA), followed by biotinylated anti-mouse and anti-rabbit secondary antibodies (R&D Systems, Minneapolis, MN, USA) and fluorescent labels (Streptavidin FITC, R&D Systems, Minneapolis, MN, USA, or Mouse IgG1 PE, R&D Systems, Minneapolis, MN, USA). After each incubation step, the cells were washed with PBS (pH = 7.4), and the containers were rotated end-over-end and centrifuged at 900× *g* for 10 min at 4 °C. A FACSCalibur^TM^ instrument with Cell Quest (Becton Dickinson, Franklin Lake, NJ, USA) software was used to analyze the quantity of cells and process intensity without sorting. Of the fixed cells, a small portion was incubated without antibodies to separate cells according to their light-scattering characteristics, and another small portion was mixed with streptavidin FITC or mouse IgG1 PE conjugates without primary antibodies to set the maximum threshold for nonspecific binding. Finally, test samples labeled with primary antibodies and fluorescent labels were scanned and analyzed. The quantity of each specific positive cell was calculated out of 50,000 total cells at maximum sensitivity.

### 4.6. Morphological Study

The brains were perfusion-fixed for further morphological studies. Visualization of the cells in the brain tissues was based on a modification of Nissl staining and Golgi silver impregnation [32], which facilitated the identification of not only large but also small neural cells. Isolated brains were fixed in 5% buffered neutral formalin for 24–48 h at 4 °C. The formalin fixative solution was prepared in 0.1 M phosphate buffer (pH 7.4) containing 0.3% CaCl_2_ and 15% sucrose. The frontal free-flow frozen slices (40–50 µm thick) were taken, washed in distilled water, and transferred into an incubation mixture containing 0.4% lead acetate, 1 M acetate buffer (pH 5.6), and 2% sodium glycerophosphate for 2–3 h at 37 °C. The slices were then washed in distilled water, transferred to a 3% sodium sulfide solution, rewashed in distilled water, and embedded into Canada balsam.

### 4.7. Behavioral Testing

Analysis of spontaneous alternation was performed using a Y maze constructed from black plastic and covered with plexiglass. After habituation, the animals were placed at the center of the maze and allowed to explore for 8 min. The maze was cleaned thoroughly between sessions. An alternation was defined as a set of three consecutive entries to alternating arms (e.g., left arm to right arm, and then back to left arm). The number of maximum alternations was calculated as the total number of arm entries minus two, and the percentage of alternation was calculated as (actual alternations/maximum alternations) × 100. To evaluate the activity and anxiety levels of animals, the elevated plus maze was used. Rats were placed on a platform consisting of two open (27 cm × 5 cm × 0.25 cm) and two closed arms (27 cm × 5 cm × 15 cm) that was elevated to a height of 40 cm above the floor. The central platform dimensions were 15 cm × 5 cm. The animals were placed on the central platform, facing the closed arm, and they were allowed to explore the elevated plus maze for 5 min [17]. Total entries to the closed and open arms and number of times that they crossed the central platform were recorded.

### 4.8. In Vivo Electrophysiology

The following electrophysiological method was used for extracellular recording and analysis of evoked activity of single neurons in the CA1 field of the hippocampus.

After 90 days of Aβ (1-42) single infusion, the animals were anesthetized with Urethan (1.1 g/kg), immobilized with 1% ditiline (25 mg/kg, intraperitoneal), fixed in a stereotaxic head frame, and placed on artificial ventilation. A stimulatory electrode was inserted according to stereotaxic coordinates [40] in the ipsilateral entorhinal cortex (EC) (AP: −9, L: ±3.5, and DV: +4.0 mm), and a glass recording electrode (1–2 μm in diameter at tip) filled with 2 M NaCl was repeatedly submerged into the hippocampal field at coordinates (AP: −3.3; L: ±1.5–3.5; DV: +3.0–4.0 mm) to record the spike activity flow of single neurons. High-frequency stimulation (HFS; 100 Hz for 1 s) was administered by means of a 0.05 ms rectangular charge that was 0.10–0.14 mA in amplitude. Recording of the spike train was carried out based on a program that selected spikes by amplitude discrimination.It helps to pinpoint spikes and excluding artifacts during HFS. The method allows to evaluate not only post-tetanic activity, but also tetanic activity. The impulse flow of neurons was subjected to software analysis, producing as output the distributed real-time pre- and post-stimulus spike activity of single neurons (Figure 7a,b). The timing, frequency, cumulative histograms, and a diagram of mean frequency for single neurons as well as populations of neurons with uniform responses were obtained. The impulse flow analysis revealed different combinations of responses: impulse flow acceleration or tetanic potentiation (TP) and post-tetanic potentiation (PTP) (TP-PTP), as well as impulse flow deceleration or tetanic depression (TD) and post-tetanic depression (PTD) (TD-PTP). The expressiveness of each response type was estimated according to the value of the mean frequency in peristimulus time (M before the event: M_be_) and post-stimulus time (M post-event: M_pe_) as well as HFS time (M high-frequency stimulation or tetaniztion time: M_tt_) (Figure 7b). Mixed combinations of responses (TD-PTP) were also recorded. In general, electrophysiological data were obtained by recording of 113 hippocampal neurons in the experimental group and 294 hippocampal neurons in the control group.

### 4.9. Statistical Analysis

Statistical analyses were performed using SPSS software (v. 26, Yerevan State Medical University named after Mkhitar Heratsi, Yerevan, Armenia). Data were checked for normal distribution using the Kolmogorov–Smirnov test. Normally distributed variables were compared with *t* tests. To increase the reliability of statistical analyses of the electrophysiological data, we also used a nonparametric method of verification by conducting the Wilcoxon rank-sum test; this method considered the asymptotic normality of this criterion and allowed for the comparison of the calculated values with the table values of the standard normal distribution. *p* values of less than 0.05 were considered statistically significant.

## 5. Conclusions

In this study, we revealed that amyloid exposure leads to the initial activation of neurogenic niches but that neural stem cells failed to complete the maturation cascade, leading to neural stem cell immaturity. Considering the abovementioned aspects, we suggest that strategies aiming to find potential biological agents, chemicals, or physical methods guiding adult neurogenesis in healthy elderly people, people at high risk of developing AD, or those who are suffering from AD both in preventive and therapeutic modes are very promising.

## Data Availability

Data will be made available by the corresponding author upon reasonable request.
